# Supplementary material for: Loss of Microglial Insulin Receptor Leads to Sex-Dependent Metabolic Disorders in Obese Mice
Source: Int J Mol Sci. 2022 Mar 8;23(6):2933. doi: 10.3390/ijms23062933 (PMC8954452; doi:10.3390/ijms23062933)
Supplement: Supplementary file 1 [file ijms-23-02933-s001.zip › ijms-1613367-supplementary.pdf]

Supplementary figures

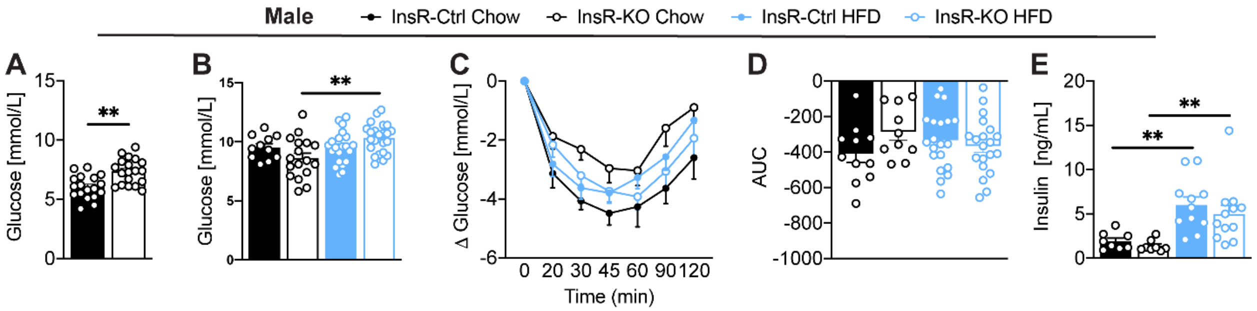

Figure S1. Loss of microglial InsR has no effect on insulin tolerance in male mice. (A-E) Male InsR-Ctrl and InsR-KO mice fed a Chow diet (black) or HFD (blue) data for (A) basal blood glucose one week after InsR deletion; (B) fasted blood glucose, following 11 weeks of diet exposure; (C-D) insulin tolerance glucose concentration, presented as (C) delta glucose and (D) AUC data; (E) fasted plasma insulin, following 11-12 weeks of diet exposure. Data are presented as mean ± SEM. **p<0.01.

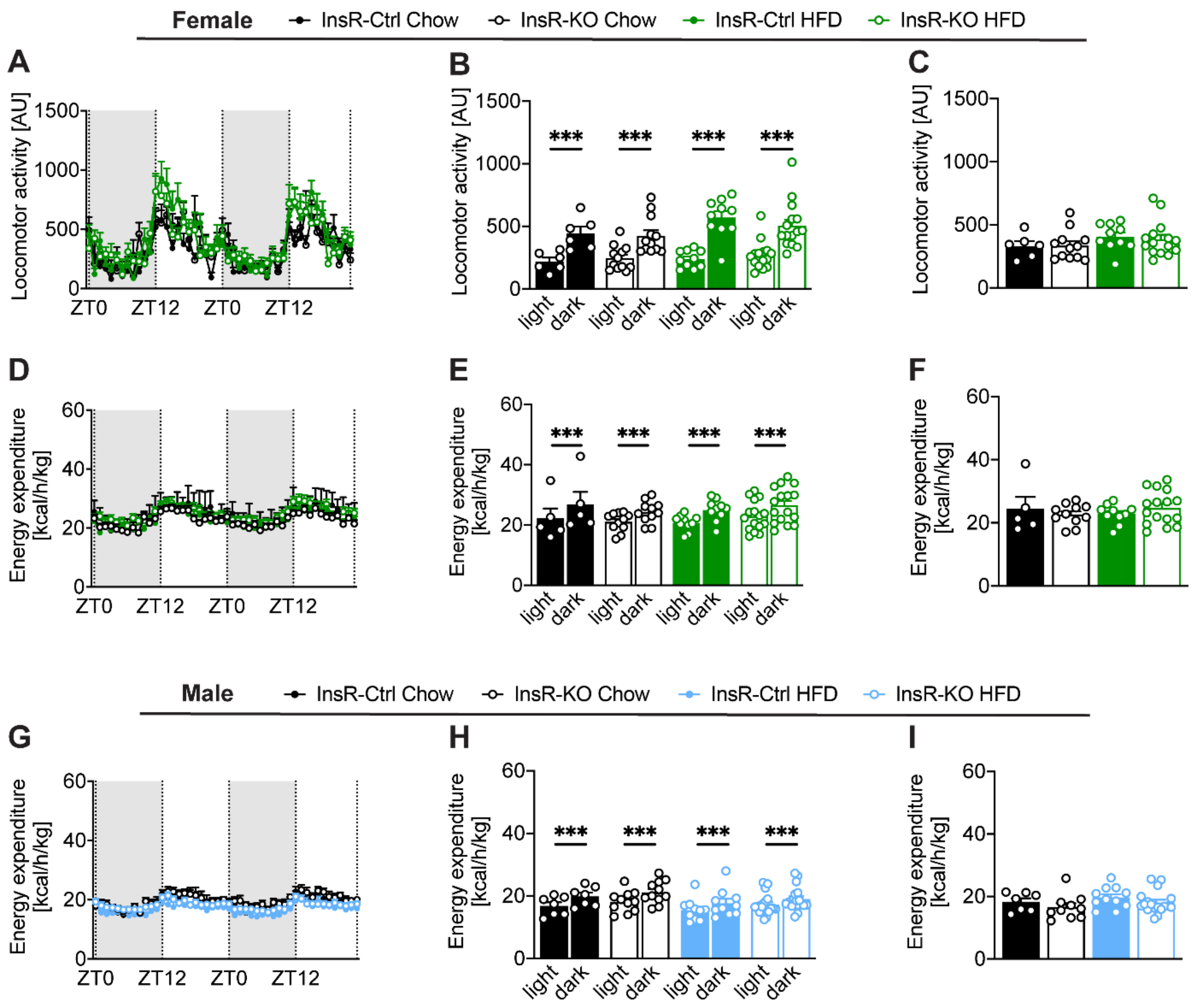

Figure S2. Loss of microglial InsR has no effect on energy expenditure in healthy and obese female and male mice. (A-F) Female InsR-Ctrl and InsR-KO mice fed a Chow diet (black) or HFD (green) data for (A-C) locomotor activity presented as (A) 48h plot, (B) 48h light and dark phase and (C) 24h; (D-F) energy expenditure data presented as (D) 48h plot, (E) 48h light and dark phase and (F) 24h. (G-I) Male InsR-Ctrl and InsR-KO mice fed a Chow diet (black) or HFD (blue) data for energy expenditure presented as (G) 48h plot, (H) 48h light and dark phase and (I) 24h. Data are measured at approximately 11 weeks of diet exposure for the duration of 48h. Data are presented as mean ± SEM. *$p<0.05$; ***$p<0.0001$.

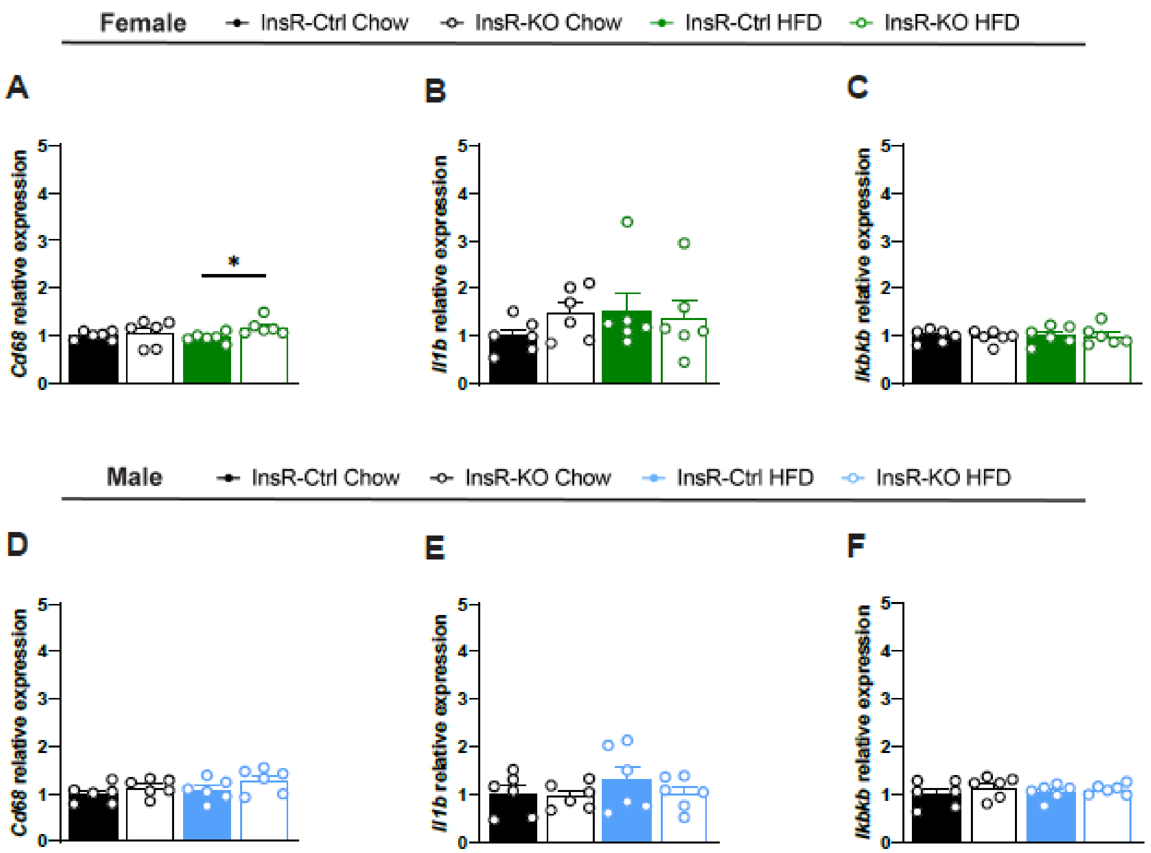

Figure S3. **Loss of microglial InsR in female mice leads to higher hypothalamic *Cd68* gene expression in obesogenic conditions.** (A-F) Relative gene expression of (A, D) *Cd68*; (B, E) *Il1b* and (C, F) *Ikbkb* from hypothalamic tissue There is an increase in *Cd68* expression in female InsR-KO mice compared to InsR-Ctrl mice under obesogenic conditions. Data presented for female InsR-Ctrl and InsR-KO mice fed a Chow diet (black) or HFD (green) and male InsR-Ctrl and InsR-KO mice fed a Chow diet (black) or HFD (blue), following 12 weeks of diet exposure. Data are presented as mean ± SEM. * $p<0.05$

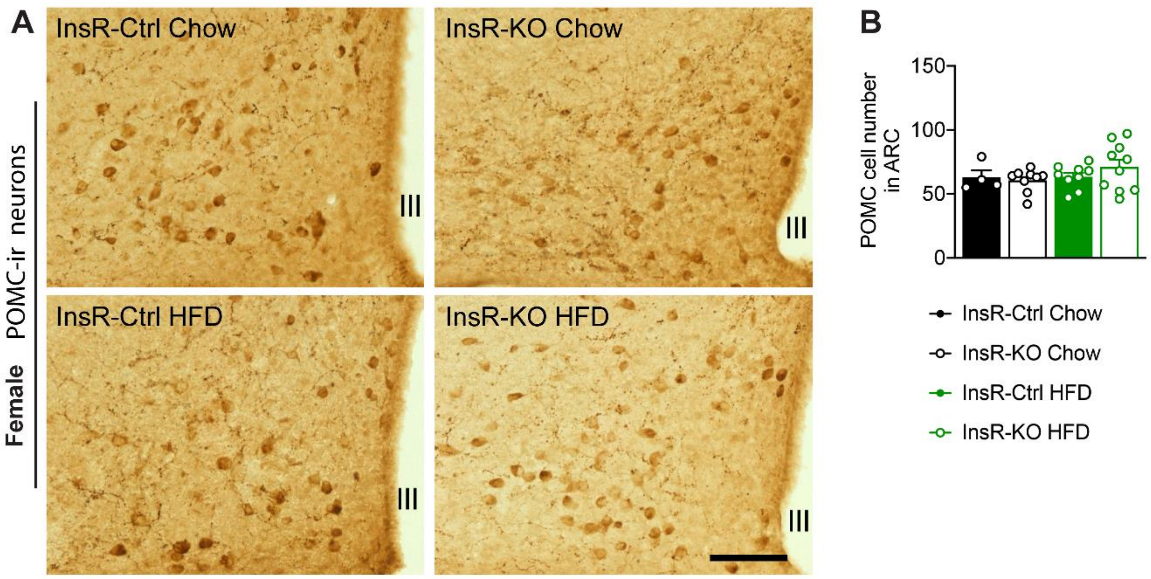

Figure S4. Lack of microglial InsR in female mice has no effect on POMC cell number in health or obesity. (A-B) POMC-ir neurons in the arcuate nucleus of female mice. Data presented for InsR-Ctrl and InsR-KO mice fed a Chow diet (black) or HFD (green), following 12 weeks of diet exposure. Data are presented as mean ± SEM. III – 3rd ventricle. Scale bar: 100 µm in (A).

Supplementary methods

Genotyping

All animals were genotyped following the suggested protocol by JAX®. Briefly, ear or toe piece is placed in 25 mM NaOH/0.2 mM EDTA solution and placed in thermocycler at 98°C for 1h. Next, we added 40 mM Tris HCL solution (pH 5.5) and we centrifuged the mix (4000 rpm, 3’). The resulting solution contains the DNA sample. We performed PCR using a PCR master mix (REDExtract-N-Amp™ PCR ReadyMix™; Ref.:R4775, Merck) and primers for the genes of interest (Cx3Cr1; InsR). PCR was performed in a thermocycler, following the guidelines of JAX®.

Primers used:

InsR forward (GGGGCAGTGAGTATTTTGGA);

InsR reverse (TGGCCGTGAAAGTTAAGAGG);

Cx3Cr1 wild type forward (AGCTCACGACTGCCTTCTTC);

Cx3Cr1 common (ACGCCCAGACTAATGGTGAC);

Cx3Cr1 mutant forward (GTTAATGACCTGCAGCCAAG).

Western blot

Primary microglial cells were snap frozen at -80°C following the 1µM 4-hydroxytamoxifen treatment. The cells were lysed in RIPA buffer (Ref: 89900, Thermo Fisher) by scraping. Protein concentration was determined by BCA assay. The samples were normalized and diluted, followed by denaturation at 95°C for 5’. Protein lysates were loaded in a 4-12% precast gel (Ref: XP04120BOX, Thermo Fisher) at concentration of 20 µg per sample, as well as an appropriate molecular weight standard (Ref: #1610374, Bio Rad). The samples were separated (LC2675, Thermo Fisher) and transferred (LC3675, Thermo Fisher) to nitrocellulose membrane. The

membranes were rinsed 3x in TBS Tween (TBS-T) solution, followed by blocking for 1h in 5% milk TBS-T solution. Primary antibodies were diluted in 5% milk TBS-T solution and incubated at for 1h at RT, followed by ON incubation at 4°C. On the next day, samples were rinsed 3x in TBS-T and incubated with a HRP-conjugated secondary antibody diluted in 5% milk TBS-T solution for 1h at RT, followed by rinsing 3x in TBS-T. The membranes were developed with ECL reagents (PRN2232, Merck), following the manufacturer's instruction, and visualized with ImageQuant LAS 4000 (GE Healthcare).

PCR

Primers used:

Hprt forward (GCAGTACAGCCCCAAAATGG);

Hprt reverse (AACAAAGTCTGGCCTGTATCCAA);

bActin forward (GCTTCTAGGCGGACTGTTACT);

bActin reverse (GCCTTCACCGTTCCAGTTTTT);

Hcrt forward (TTCCTTCTACAAAGGTTCCCTG);

Hcrt reverse (TGGTTACCGTTGGCCTGAAG);

Agrp forward (AACTCTGACCAAATCCACCCC);

Agrp reverse (TGAGGTGCCTCCCATTGTGT);

Npy forward (CCCGCCACGATGCTAGG);

Npy reverse (TGATGTAGTGTCGCAGAGCG);

Cd68 forward (CTAGGACCGCTTATAGCCCAAG);

Cd68 reverse (TGCCATTTGTGGTGGGAGAA);

Il1b forward (TGCCACCTTTTGACAGTGATG);

Il1b reverse (TGATGTGCTGCTGCGAGATT);

Ikbkb forward (TACCCTGCAGGAAGCTACATC);

Ikbkb reverse (TTGTGTGAGCATCTCTTCGCT);

Immunohistochemical and fluorescent stainings

Briefly, mice were injected with 50 ul pentobarbital for every 10g of BW. Mice were perfused intracardially and fixed with 4% paraformaldehyde (solved in PBS, pH 7.4) at 4°C. Brains were incubated at 4°C on a shaking platform in 30% sucrose solution (solved in 1x TBS and 0.05% sodium azide) for approximately 48h until sunk at the bottom. 30 um coronal sections were sliced on a cryostat and the sections were rinsed in TBS. The sections were incubated with 10% Methanol-10% Hydrogen peroxide solution (in TBS) for 10' followed by rinsing 3x in TBS. The brain slices were incubated with primary antibodies solved in SUMI solution (0.625g gelatin, 1.25mL TritonX-100 in 250 mL TBS) at RT for one hour, followed by incubation at 4°C overnight. Sections were rinsed 3x in TBS and incubated in biotinylated secondary antibody solved in SUMI for 1h at RT. The sections were rinsed 3x in TBS and incubated in avidin-biotin complex (Ref: PK-6100, Vector Laboratories). The reaction product was visualized by incubation in 1% diaminobenzidine with 0.01% hydrogen peroxide.
